# Probabilistic model checking of cancer metabolism

**DOI:** 10.1038/s41598-022-21846-5

**Published:** 2022-11-07

**Authors:** Meir D. Friedenberg, Adrian Lita, Mark R. Gilbert, Mioara Larion, Orieta Celiku

**Affiliations:** 1 Cornell University, Ithaca, NY 14850 USA; 2 National Cancer Institute, Bethesda, MD 20892 USA

**Keywords:** Cancer metabolism, Cancer models, CNS cancer

## Abstract

Cancer cell metabolism is often deregulated as a result of adaption to meeting energy and biosynthesis demands of rapid growth or direct mutation of key metabolic enzymes. Better understanding of such deregulation can provide new insights on targetable vulnerabilities, but is complicated by the difficulty in probing cell metabolism at different levels of resolution and under different experimental conditions. We construct computational models of glucose and glutamine metabolism with focus on the effect of IDH1/2-mutations in cancer using a combination of experimental metabolic flux data and patient-derived gene expression data. Our models demonstrate the potential of computational exploration to reveal biologic behavior: they show that an exogenously-mutated IDH1 experimental model utilizes glutamine as an alternative carbon source for lactate production under hypoxia, but does not fully-recapitulate the patient phenotype under normoxia. We also demonstrate the utility of using gene expression data as a proxy for relative differences in metabolic activity. We use the approach of probabilistic model checking and the freely-available Probabilistic Symbolic Model Checker to construct and reason about model behavior.

## Introduction

Deregulation of cell metabolism has recently been designated an emerging hallmark of cancer^1^. This designation recognizes both the important role that reprogramming of energy metabolism plays in cancer, as well as the need to better understand the mechanisms by which this reprogramming is achieved, its relation to other signaling-related hallmarks, and ultimately how it can be exploited to develop new therapies.

The classical cancer metabolic phenotype, known as the Warburg phenotype^2^, is characterized by a shift toward relying on aerobic glycolysis—an inefficient way of metabolizing glucose to supply cellular energetics that is typically deployed in hypoxic conditions. The Warburg phenotype has been observed in many aggressive cancers, and is thought to be an adaptation that enables fast-growing cells to fuel biosynthesis through glycolytic intermediates, and thrive in the tumor micro-environment characterized by gradients of hypoxia.

In some cancers metabolism is directly affected through mutation of key metabolic enzymes, most notably the IDH family of enzymes. IDH1, IDH2, and IDH3 catalyze the conversion of (iso)citrate to $\alpha$-ketogluterate ($\mathrm {aKG}$) in the cytoplasm (IDH1) and mitochondria (IDH2 and IDH3). Single amino acid missense mutations in IDH1 at arginine 132 (R132) or the analogous residue in IDH2 (R172 or R140) lead to gain of novel catalytic function—the conversion of $\mathrm {aKG}$ to (D)2-hydroxyglutarate ($\mathrm {2HG}$)^3^. These mutations are found in more than 80% of lower grade gliomas and secondary glioblastomas, as well as in acute myeloid leukemia, myelodysplastic syndromes and myeloproliferative neoplasms, and cholangiocarcinoma^4,5^. The accumulation of $\mathrm {2HG}$, which has no physiologic function in mammals, has an oncogenic effect through a number of mechanisms, which include epigenetic silencing of transcription. IDH mutation status has also been shown to have prognostic value: patients with IDH-mutant gliomas live longer than those with IDH-wild-type gliomas, even when controlling for other prognosticators like age at diagnosis and grade.

The discovery of the oncogenic role of $\mathrm {2HG}$ fueled hopes that inhibiting mutant IDH and therefore its ability to produce $\mathrm {2HG}$ would be an effective therapy against IDH-mutant cancers. However, inhibition of the mutant IDH function has not translated into better outcomes. These failed efforts might be due to the high levels of $\mathrm {2HG}$ playing an initiator role in cancer genesis (for example, IDH mutations in gliomas have been shown to proceed TP53 mutations or 1p/19q chromosome deletions) that once started becomes independent of the levels of $\mathrm {2HG}$. However, other consequences of IDH mutations have been discovered: for example, recent studies have shown that the mutations compromise the ability of wild-type IDH to catalyze the reverse conversion of $\mathrm {aKG}$ or glutamate to citrate^6–8^.

Many unanswered questions remain on the role of IDH mutations in cancer. For example, while typically slow growing, IDH-mutant gliomas eventually progress to an aggressive phenotype with Warburg-like metabolism^9^. How these metabolically-defective cells adapt to the high energy demands of fast-growing cells is not known. The feedback loop between the microenvironment pressures (such as hypoxia or nutrient scarcity) and the shift in metabolic phenotypes is not well-understood.

Advances in technologies for profiling the metabolome are making it possible to glimpse at the cancer metabolic profiles both in a wide range of biological models as well as directly in patients. Metabolic Flux Analysis (MFA), for example, can be used to determine the flux of molecules in a metabolic pathway through tracing of quantities of ($^{13}C$-)labeled metabolites^10^. Next-generation sequencing, mass spectrometry and other high-throughput assaying technologies are used to determine the quantities of gene-products and infer pathway activity. However, assaying the metabolic profiles under varying, physiologically-relevant conditions, and integrating the obtained information remains difficult.

Systems biology approaches that marry pragmatic modeling to theoretical exploration can markedly enhance the technological advancement and bridge the information gap^11^. “Executable biology”—computational models that include a recipe for an abstract execution engine—can be constructed with incomplete information and used to reason about complex chains of events that mimic natural phenomena^12^. Crucially, such models can be tested under assumptions that might be experimentally impractical to recapitulate. For example, the behavior of the system can be studied under different assumptions on nutrient availability, which can help with generating experimental hypotheses, and prioritizing experiments.

In this article, we report on the use of probabilistic model checking, an approach developed for modeling and reasoning about systems with inherent stochastic behavior, to model and reason about glucose and glutamine metabolism in cancer and how it is affected by mutations in IDH. Specifically, we use the Probabilistic Symbolic Model Checker (PRISM)^13^, an integrated environment that provides a high-level modeling language, a temporal-property specification language, and an associated tool for automatic property verification. The models are initially constructed using MFA data from Grassian et al.^14^, who profiled colorectal carcinoma cell line models that differ only in their IDH-mutation status under normal and low-oxygen conditions. Our approach provides a global view of metabolism and highlights the differences between normoxia and hypoxia. In a further step, we integrate MFA data with patient-derived gene expression data from The Cancer Genome Atlas (TCGA) (http://cancergenome.nih.gov/) to predict the metabolic profiles of normal brain and IDH-mutant gliomas. Surprisingly, the models derived from patient data point to differences due to IDH-mutation status that are not recapitulated in the experimental models. The model is publicly available in the Biomodels repository with MODEL22071800001 identifier.

## Methods

A metabolic pathway is a set of biochemical reactions that transform substrates into products in the presence of enzymes. Each reaction is characterized by a rate, which depends on the concentration of the substrates and a rate constant dependent on the characteristic of the enzymes catalyzing the reaction.

Chemical reactions can be described following the law of conservation of mass. For example, the first reaction of glycolysis—the conversion of one molecule of glucose to one molecule of glucose-6-phosphate—is captured by this simple expression of the law:

1 $$\begin{aligned} \mathrm {Gluc}+ \mathrm {ATP}\xrightarrow {\mathrm {HK}}_k \mathrm {G6P}+ \mathrm {ADP}\end{aligned}$$

where $\mathrm {HK}$ is the enzyme catalyzing the reaction, and *k* is the reaction constant. The reaction also requires one molecule of $\mathrm {ATP}$, which is subsequently transformed into one molecule of $\mathrm {ADP}$.

Enzyme kinetics are often complex and dependent on the experimental conditions (temperature, volume, pressure) and relative concentrations of enzymes and substrates. However, a frequently-used simplifying assumption, known as the law of mass action is that the rate of reactions is proportional to the concentration of substrates and the reaction constant.

We follow Kwiatkowska et al.’s approach^15,16^ to modeling molecular entities and reactions as computer processes. The molecular entities are modeled as discrete variables, and the reactions as guarded actions of the form:

2 $$\begin{aligned}{}[\mathrm {action}]~\mathrm {guard} \rightarrow \mathrm {rate}: \mathrm {update} \end{aligned}$$

where $\mathrm {guard}$ expresses the conditions under which the reaction is possible, $\mathrm {rate}$ is the rate of the reaction, and $\mathrm {update}$ describes how the quantities of substrates and products are transformed by the reaction. $\mathrm {Action}$ is an optional action label. Unless otherwise stated, we will abstract over which enzymes are responsible for catalyzing the reaction; other abstractions may include leaving out molecules whose behavior is not directly relevant to the properties that we seek to explore. We will also assume that the rate of the reaction depends only on the reaction constant and the concentration of the substrates.

The first reaction of glycolysis at (1) can thus be modeled as:

3 $$  \begin{aligned} \begin{gathered} []~\overbrace{\mathrm {Gluc}> 0 ~ \& ~ \mathrm {G6P}< \mathrm {G6P}_\mathrm {max}}^{\mathrm {guard}} \rightarrow \overbrace{R_1 * \mathrm {Gluc}}^{\mathrm {rate}}: \overbrace{\mathrm {Gluc}' = \mathrm {Gluc}- 1~ \& ~ \mathrm {G6P}' = \mathrm {G6P}+ 1}^{\mathrm {update}} \end{gathered} \end{aligned}$$

It states that the reaction is possible when the number of glucose molecules is nonzero, and glucose-6-phosphate is not saturated. The rate of the reaction in a given state is proportional to the number of glucose molecules, and the reaction constant $R_1$. When the reaction is executed, it leads to a new state in which the number of glucose molecules is reduced by 1 and the number of glucose-6-phosphate molecules is increased by one. This representation abstracts over the $\mathrm {ATP}$-dependence of the reaction.

Each reaction can thus be modeled as a guarded command. The set of resulting guarded commands is executed in parallel: in particular, when two or more reactions are enabled, a race between the enabled reactions ensues, and the first to be triggered determines how the variables are updated. The reactions that share a label are executed synchronously, as one joint command: their guards must all hold for the command to be enabled, the rates are multiplied, and the updates take place simultaneously. The language also provides mechanisms that enable modular specifications.

Properties that can be specified over such models are temporal and quantitative in nature. Some examples are: “What is the probability of reaching a state in which lactate is depleted/saturated?” “What is the quantity of glucose at a given instant in time?” and “How many glucose-to-glucose6-phosphate reactions happen on average over a period of time?”

### Metabolism and experimental background

Our focus is on modeling glycolysis and the tricarboxylic acid (TCA) cycle and how they are affected by mutations in IDH. We abstract over the compartment where the reactions happen and therefore assume that $\mathrm {aKG}$ is available for conversion to $\mathrm {2HG}$ when either IDH1 or IDH2 are mutated, which we collectively denote as $\mathrm {IDH}^\mathrm {mut}$.

#### Metabolic flux analysis

Grassian et al.^14^ studied the metabolic phenotype of cell lines that differ only with respect to their IDH1-mutation status. $^{13}C$ MFA was performed to estimate the flux through a metabolic network comprising of glycolysis, the pentose phosphate pathway (PPP), the TCA cycle, biomass synthesis, and fatty acid synthesis. HCT116 colorectal carcinoma cells with wild type IDH1 (parental) or heterozygous IDH1 R132H/+ were studied under normoxia (21% $\mathrm {O_2}$) or hypoxia (2% $\mathrm {O_2}$) conditions.

The flux rates from Grassian et al.^14^ were used to instantiate our model of glycolyis and TCA with four phenotypes: $\mathrm {IDH}^\mathrm {wt}$ and $\mathrm {IDH}^\mathrm {mut}$ under normoxia and hypoxia. All the rates were normalized to the rate of the first glycolysis reaction of the $\mathrm {IDH}^\mathrm {wt}$ phenotype. For model reactions that represent a chain of measured reactions we use the minimum rate, representing the rate limiting step of the chain.

#### Patient-derived expression profiles

The $\mathrm {IDH}^\mathrm {wt}$ phenotype as instantiated with the MFA rates from the normoxia experiments was used in conjunction with patient-derived mRNA expression data to predict the metabolic phenotypes of normal brain (at the whole organ level), and $\mathrm {IDH}^\mathrm {mut}$ gliomas. We follow the approach of Khurshed et al.^17^, who used relative expression of genes that code for key metabolic enzymes to estimate the relative rates of key metabolic reactions.

RNA-sequencing data from 665 gliomas with known IDH-mutation status, and 5 normal brain patient samples generated from The Cancer Genome Atlas (TCGA) Research Network (https://cancergenome.nih.gov/)^18^ were downloaded and processed using R’s^19^ Bioconductor^20^ package TCGAWorkflow^21^. The samples were split into cohorts as follows: 140 glioblastomas (GBMs) with wild type IDH formed the Warburg phenotype; 419 lower grade gliomas (LGGs) with mutant IDH formed the $\mathrm {IDH}^\mathrm {mut}$ phenotype; the 5 normal samples formed the Normal phenotype. The remaining samples were considered representatives of intermediate phenotypes and were excluded from the analysis.

Kaplan–Meier survival of the glioma cohorts were performed using R’s survival package.

Preprocessing, normalization, and filtering of the raw transcript counts were performed as recommended in TCGAWorkflow^21^. Default recommended settings were used to perform differential gene expression analysis for each of the pair-wise comparisons of the phenotypes of interest and to determine the fold change (ratio) of the gene expression.

The fold changes of isoenzymes—enzymes catalyzing the same reaction—were aggregated by averaging to obtain a single fold change for the corresponding reaction. The flux rates for the Normal phenotype were computed by dividing the flux rates of the Warburg phenotype ($\mathrm {IDH}^\mathrm {wt}$ phenotype from MFA), with the fold changes $\frac{{\text {Warburg}}}{{\text {Normal}}}$ of the enzymatic group catalyzing the corresponding reaction. The $\mathrm {IDH}^\mathrm {mut}$ phenotype was computed by dividing the Warburg rates with the $\frac{\text {Warburg}}{\mathrm {IDH}^\mathrm {mut}}$ fold changes. The rate for conversion of $\mathrm {aKG}$ to $\mathrm {2HG}$ for the $\mathrm {IDH}^\mathrm {mut}$ phenotype was taken from the corresponding rate of Grassian et al.^14^ of the normoxia $\mathrm {IDH}^\mathrm {mut}$ phenotype.

### Computational approach

PRISM^13^ can be used for specification and verification of systems in which transitions from one state to another happen stochastically. We briefly describe the semantics of PRISM models, the logic used to express the properties, and the intuition behind probabilistic model checking following^16,22^. We refer the reader to the literature for a rich set of publications that detail the semantics and the model checking technique further.

#### Model semantics and property specification

The semantics of guarded commands is based on Continuous Time Markov Chains (CTMCs), which are extensions of Discrete Time Markov Chains (DTMCs). DTMCs model events that happen in lockstep in discrete steps, whereas CTMCs model events that happen in continuous real time, with the time required to make a transition being a random variable drawn from an exponential distribution.

##### Definition

A Discrete-Time Markov Chain (DTMC) is a tuple ${\mathscr {D}}=(S, {\overline{s}},P,AP,L)$, where *S* is a finite set of states, ${\overline{s}}\in S$ is a distinguished initial state, $P:S\times S \rightarrow [0,1]$ is a transition probability matrix such that $\sum _{s'\in S} P(s, s')=1$ for all $s \in S$, *AP* is a set of atomic propositions, and $L(s) \subseteq AP$ is a labeling function.

In a DTMC model, transitions between states are denoted by discrete probability distributions, which represent the likelihood of moving to a target state. The behavior of a DTMC is the set of the execution paths $s_0s_1s_2\ldots$ such that $s_0$ is the initial state and $P(s_i, s_{i+1}) > 0$ for all *i*. The Markovian assumption, which states that $P(s_i, s_{i+1})$ depends only on $s_i$, and not the paths that were taken to reach it, gives the model enough structure and simplicity to allow for computations of complex properties over the behavior of DTMCs.

##### Definition

A Continuous-Time Markov Chain (CTMC) is a tuple ${\mathscr {C}} = (S; {\overline{s}};R;AP,L)$ where *S*, ${\overline{s}}$, *AP*, and *L* are defined as for DTMCs and $R:S \times S \rightarrow \mathbb {R}_{\ge 0}$ is the transition rate matrix.

$R(s, s')$ specifies the rate of transitioning from state *s* to $s'$. A transition can only occur between states *s* and $s'$ if $R(s, s')>0$, and the probability of this transition being triggered within *t* time units is $1-e^{-R(s,s')\cdot t}$. When two or more transitions from state *s* have positive rates, the first transition to be triggered determines the next state of the CTMC. A path of *C* is a finite or infinite sequence $s_0t_0s_1t_1s_2 \ldots t_{n-1}s_n$, where $t_i \in \mathbb {R}_{>0}$ for each $i\ge 0$ represents the amount of time spent in state $s_i$.

The exit rate from a state *s* is the sum of the rates of all the transitions leaving from *s*, $E(s) = \sum _{s'\ne s} R(s,s')$; the time spent in *s* before a transition is exponentially distributed in *E*(*s*). A transition probability matrix can be defined such that

4 $$\begin{aligned} P(s,s')={\left\{ \begin{array}{ll} R(s,s')/E(s), &{} \text {if}\;E(s)\ne 0\\ 1 &{} \hbox {if}\; E(s) = 0\; \hbox {and}\; s = s'\\ 0 &{} \text {otherwise} \end{array}\right. } \end{aligned}$$

The resulting DTMC ${\mathscr {D}} = (S,{\overline{s}},P, AP, L)$ is said to be embedded in ${\mathscr {C}}$. In settings like ours, where we model reactions happening at different but specified rates and no true nondeterminism is modeled, CTMCs are the natural modeling choice.

Properties are expressed in some flavor of probabilistic temporal logic, for example, Probabilistic Computation Tree Logic (PCTL)^23^, or continuous-time formulations, for example, Continuous Stochastic Logic (CSL)^24^. The properties that can be specified fall under two categories: state properties, denoted with $\Phi$, or path properties, denoted with $\Psi$. State property operators combine simple atomic propositions and logical operators such as conjunction and disjunction, and are interpreted building up from the interpretation of simple atomic propositions—a state $s\in S$ satisfies an atomic proposition *a* if $a\in L(s)$—to the interpretation of the rest of the logical constructs. Moreover, CSL includes a steady state operator *S*: a steady state property ${\mathrm{S}_{\ge \mathrm{p}}} \Phi$ is satisfied if the probability of $\Phi$ holding in steady state is $\ge p$.

PCTL and CSL also contain a probabilistic state operator which operates on path formulas. The probabilistic formulas are interpreted as follows: a state *s* satisfies ${\mathrm{P}_{\ge \mathrm{p}} \Psi }$, for example, if the probability that the path leading from *s* satisfies $\Psi$ is $\ge p$. Path formulas are constructed with operators next and until. For example, the next operator  ${\mathrm{P}_{\ge \mathrm{p}} \mathrm {X} \Phi }$ is satisfied in state *s* if with probability greater than or equal to *p* the next state we transition to from *s* will satisfy $\Phi$.

Quantitative versions of the properties, denoted as $\mathrm {P}=_?[\Psi ]$ enable asking directly what the probability of satisfying $\Psi$ is. A further generalization, the extension of the logics with a reward operator, enables computing the expected value of reward structures, which are defined over states or transitions and range over $\mathbb {R}_{\ge 0}$. Examples of properties that can be expressed and are relevant to our context include:

- $\mathrm {P} =_? [ true~\mathrm {U}^{\le 15} (\mathrm {Lac}=\mathrm {Lac}_\mathrm {max})]$—“What is the probability of reaching a state in which lactate is saturated within 15 steps?”
- $\mathrm {R}[``\mathrm {Cit}'']=_?[\mathrm {I}=10]$—“What is the amount of citrate at time point 10?”
- $\mathrm {R}[``\hbox {{Pyr}-to-Lac}'']=_?[\mathrm {C}^{\le 100}]$—“What is the expected total number of $\mathrm {Pyr}$-to-$\mathrm {Lac}$ reactions performed within 100 time steps?”

#### Model checking

The problem of model checking is that of deciding if a DTMC/CTMC model satisfies a property expressed as a PCTL/CSL formula. The model checking problem for CTMCs is typically transformed into the simpler problem of model checking of the embedded DTMCs. For the rest of the present paper, the underlying models are CTMCs.

Efficient methods for automatic model checking of temporal logics and their probabilistic variants have been developed using a combination of graph algorithms, fixpoint computations, and techniques for computing optimal solutions of systems of equations. For very large models for which model checking is infeasible, their behavior can be explored through simulation or statistical model checking: generating a large number of random paths through the model, evaluating the result of the given properties for the sampled paths, and using the information to estimate approximately correct results^25,26^.

#### PRISM

Command-line PRISM^13^ was used for model checking. Results were plotted using Python’s matplotlib library. Scipy’s interpolation functionality was used to perform smoothing on the 3D plots.

## Results

### Model construction

**Figure 1:**
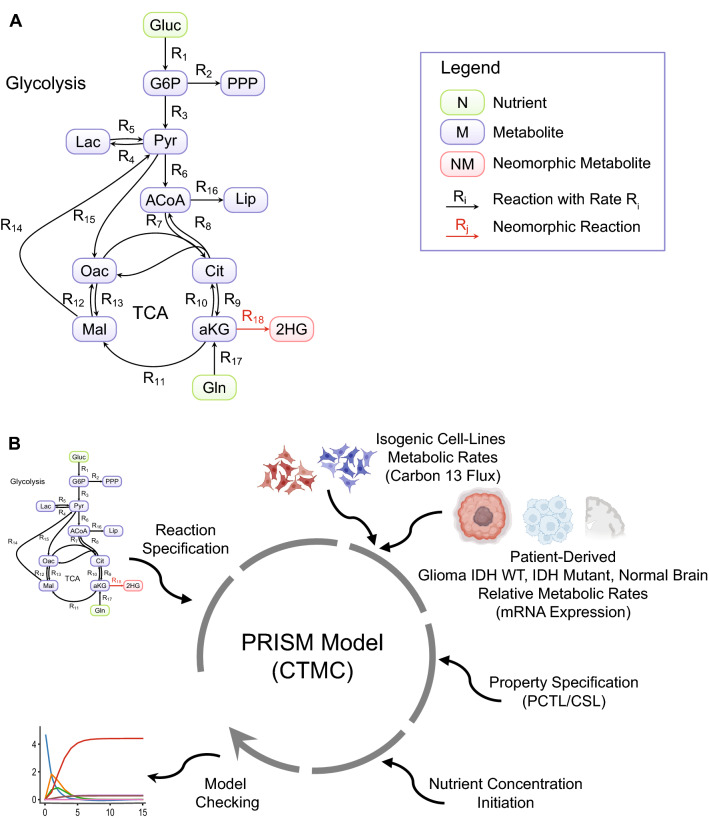
Modeled pathway and model creation workflow. (**A**) Glycolysis and TCA cycle reactions modeled, including neomorphic activity of IDH Mutant. For glycolysis the following metabolites and abbreviations were used: Gluc, glucose; G6P, glucose-6-phosphate; Pyr, pyruvate; Lac, lactate; ACoA, acetyl-CoA; PPP, pentose phosphate pathway; Lip, lipids. For TCA cycle the following metabolites and abbreviations were used: Gln, glutamine; Cit, citrate; aKG, alpha-ketoglutarate; Mal, malate; Oac, oxaloacetate; 2HG, 2-hydroxyglutarate. (**B**) Workflow of PRISM model creation: specification of reactions; instantiation of rates with Grassian et al.^14^ metabolic flux rates of isogenic colorectal carcinoma cell lines (with the parental cell line have IDH wild type, and a derived cell line with induction of IDH mutation expression) under normoxia and hypoxia; use of TCGA mRNA expression from patient derived glioma (IDH WT and IDH Mutant) and normal brain to estimate relative rates of metabolic activity for the pairs; specification of properties in probabilistic logic; instantiation of nutrient concentration; model checking of resulting models.

In this study, we constructed a model of a simplified metabolic network consisting of components from glycolysis, glutamine metabolism, TCA cycle, and several reactions feeding into PPP, and fatty acid synthesis. The resulting pathway diagram is depicted in Fig. 1A. We have abstracted over cell-compartment information and therefore the need to model shuttling of molecules in and out of them.

The model construction workflow is depicted in Fig. 1B. Briefly, the steps for constructing and reasoning about the models consist of (1) specifying the reactions of the pathway, (2) instantiating the rates of the reactions with experimental or a mix of experimental and patient-derived data as will be elaborated below, (3) specifying the properties that we are interested in reasoning about, (4) initiating the quantities of the system nutrients, and (5) performing the model checking of the properties and displaying the results.

The same model can be used to study the behavior of different phenotypes, based on how the reaction rates are instantiated. The unique ability of $\mathrm {IDH}^\mathrm {mut}$ to catalyze the novel conversion of $\mathrm {aKG}$ to $\mathrm {2HG}$ reaction is captured in the model by conditioning the execution of the corresponding guarded command on the existence of $\mathrm {IDH}^\mathrm {mut}$ molecules. We assume that all molecules have a maximum amount that they can reach, beyond which they are considered saturated and any reaction producing more of them is disabled.

We study the behavior of phenotypes by model checking several types of properties on the amount of molecular quantities over time including computing transient amounts (which give snapshots of the pathway at instants in time), cumulative amounts over intervals of time, as well as the probability of reaching certain qualitative states. This is possible due to the expressiveness of the property specification language based on PCTl/CSL and the model checking machinery, which enables their automatic verification or simulation. Moreover, the PRISM framework also allows model checking of these properties under different initial concentrations enabling reasoning about how these transient amounts and cumulative amounts change as a function of nutrient concentration.

### Isogenic cell lines display classical Warburg phenotype

We instantiated our metabolic model with MFA data published by Grassian et al.^14^, who profiled isogenic colorectal carcinoma cell lines (that differed only on the IDH-mutation status) under normoxia and hypoxia. To our knowledge, this is the only publicly available dataset derived from direct measures of metabolic fluxes from $\mathrm {IDH}^\mathrm {wt}$ and $\mathrm {IDH}^\mathrm {mut}$ cell lines, and which includes rates measured both under normoxia and hypoxia. Therefore, despite the cell lines not being glioma-derived, we chose these rates as the starting point. The raw rates, given in $\mathrm {fmol}/\mathrm {cell}/\mathrm {hour}$, were normalized to the rate of the first glycolytic reaction of the parental $\mathrm {IDH}^\mathrm {wt}$ cell line under normoxia, which was measured as 302$\mathrm {fmol}/\mathrm {cell}/\mathrm {hour}$. (The normalized MFA rates are shown in Supplemental Table S1.)

**Figure 2:**
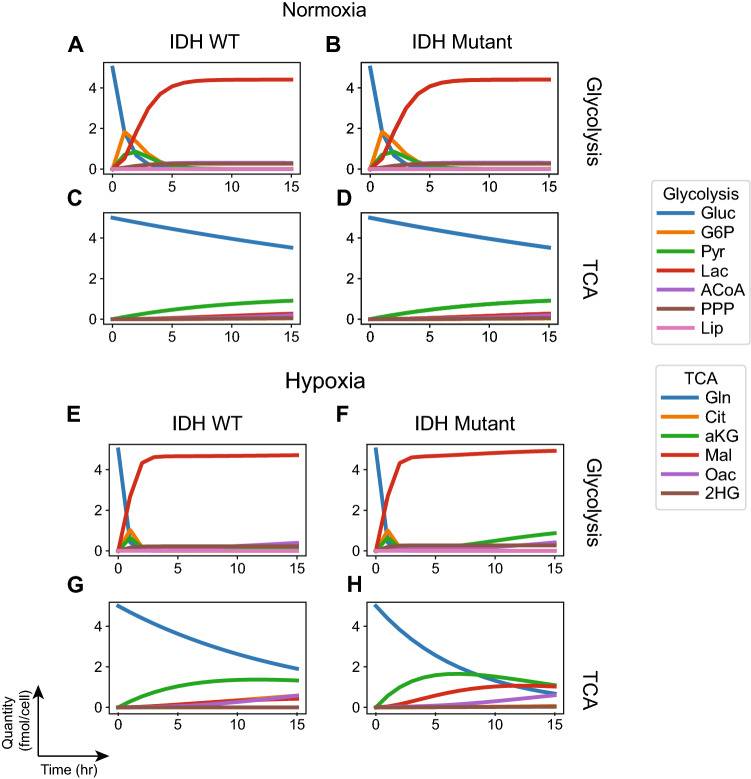
Metabolic phenotypes based on flux rates measured on isogenic cell lines. (**A**–**H**) Rates from Grassian et al.^14^ were used to instantiate the models of behavior under normoxia (**A**–**D**) and hypoxia (**E**–**H**). The model checking problem was expressed as characterizing the transient quantity of molecular species at a given instant in time. Glucose and glutamine are initiated at maximum quantity 5 (fmol/cell) and all other species at 0.

We studied the behavior of the phenotypes of the $\mathrm {IDH}^\mathrm {wt}$ and $\mathrm {IDH}^\mathrm {mut}$ cell lines under normoxia and hypoxia by computing the amount of molecular quantities over time. The findings shown in Fig. 2 point to rapid utilization of glucose in both cell lines. Interestingly, both cell lines produce high levels of lactate. TCA activity is by comparison slow, with glutamine utilization, being slower. These phenotypes resemble the classical Warburg phenotype.

The $\mathrm {IDH}^\mathrm {wt}$ cell line profiled by Grassian et al.^14^ is a patient-derived cell line, whereas the $\mathrm {IDH}^\mathrm {mut}$ counterpart was obtained through experimentally-induced overexpression of mutant IDH1 in it. Our findings suggest that the introduction of mutant IDH in a Warburg-like cell line does not change the phenotype significantly. Nevertheless, small amounts of $\mathrm {2HG}$ are produced by the derived cell line confirming the gain of function of $\mathrm {IDH}^\mathrm {mut}$.

### Model validation

Another appeal of utilizing Grassian et al.’s^14^ MFA data was that the authors of the study applied standard deterministic kinetic approach to modeling, the preferred mathematical modeling approach for metabolic pathways. Deterministic kinetic models are based on Ordinary Differential Equations (ODEs), which can be used to describe the change in molecular concentrations, and are modeled as continuous functions. The systems of ODEs describing a molecular network can be explored through mathematical analysis, when the constraints on them are simple, or through computational simulation of how the quantities change over time while satisfying any imposed constraints. Grassian et al.’s work therefore enabled us to compare and validate our results against the deterministic kinetic approach.

We reproduced the trends observed by Grassian et al., in both the effect of hypoxia on parental $\mathrm {IDH}^\mathrm {wt}$ cells as well as the effect of introducing $\mathrm {IDH}^\mathrm {mut}$ into the parental cells. Specifically, we show that our model recapitulates the faster utilization of glucose (Fig. 2A vs. E, blue line) (Fig. 2B vs. F, blue line) and glutamine (Fig. 2C vs. G, blue line) (Fig. 2D vs. H, blue line) by both cell lines in hypoxia.

Grassian et al.^14^ normalized the $\mathrm {IDH}^\mathrm {mut}$ flux to the $\mathrm {IDH}^\mathrm {wt}$ in hypoxia and reported the ratios between these two conditions. They found significant changes in hypoxia, where the mutant increased the flux of glutamine utilization into $\mathrm {aKG}$, fumarate, malate, and pyruvate, known as the oxidative part of the TCA cycle. Our model reported the same findings (Fig. 2, TCA cycle, hypoxia). Since we have reported the $\mathrm {IDH}^\mathrm {wt}$ and $\mathrm {IDH}^\mathrm {mut}$ separately, we also need to compare the rates of glutamine consumption in hypoxia. Indeed, glutamine utilization is faster in $\mathrm {IDH}^\mathrm {mut}$ as seen by the faster decay of the blue line in Fig. 2H compared with Fig. 2G (glutamine utilization). In addition, $\mathrm {aKG}$ production is faster and higher in amplitude in the $\mathrm {IDH}^\mathrm {mut}$ compared with the $\mathrm {IDH}^\mathrm {wt}$ (Fig. 2H compared with Fig. 2G, green line), malate production is faster (red line in Fig. 2H vs. G). These results suggest, that $\mathrm {IDH}^\mathrm {mut}$ cell lines depend on glutamine for the oxidative TCA cycle more than the $\mathrm {IDH}^\mathrm {wt}$ counterpart, which was also reported by by Grassian et al.^14^.

### Hypoxia increases dependence on glutamine as alternative carbon source

Hypoxia induces faster rates of nutrient utilization in both cell lines (Fig. 2). The differences with the normoxia phenotypes are especially pronounced for the $\mathrm {IDH}^\mathrm {mut}$ derived cell line: glutamine utilization is faster, and the flux through oxidative TCA cycle (reactions from $\mathrm {aKG}$ to $\mathrm {Oac}$) is accelerated.

**Figure 3:**
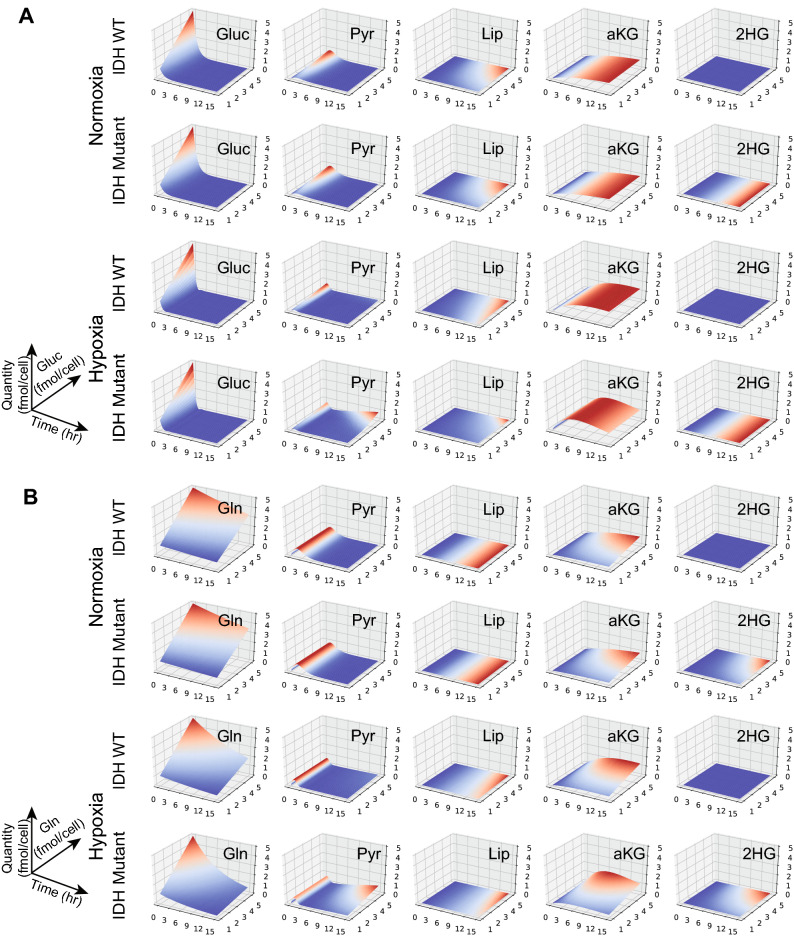
Effects of varying nutrient concentration on metabolites under normoxia and hypoxia conditions. (**A**) Effect of varying inital glucose concentration (from 1 to 5) with initial glutamine set to 5 (fmol/cell). (**B**) Effect of varying initial glutamine concentration (from 1 to 5) with initial glucose set to 5 (fmol/cell). The PRISM models were instantiated with corresponding phenotype and condition rates as in the experimental models. All other molecular species were initialized with 0. The model checking problem was expressed as characterizing the transient quantity of molecular species at a given instant in time.

The dependence on glucose and glutamine as carbon sources is better understood through experiments that study the effect of varying initial concentrations of nutrients on the transient amounts and cumulative quantities of the metabolites. The results are shown in Fig. 3 for transient properties (and in Supplemental Figure S1 for cumulative properties). These show that under normoxia for both phenotypes, the glycolytic intermediates are derived from glucose—the amounts of the intermediates increase with the increase in glucose concentration but are not affected by the glutamine concentration. The TCA intermediates are mostly derived from glutamine—the amounts of intermediates increase with the increase in glutamine concentration but are not affected by the glucose concentration.

Hypoxia conditions lead to notable differences in dependence on glutamine: $\mathrm {ACoA}$ and $\mathrm {Lip}$ production now also depend on the concentration of glutamine in addition to the concentration of glucose. Most notably for the $\mathrm {IDH}^\mathrm {mut}$ cell line, the flux of metabolites derived from TCA leads to pyruvate, and ultimately lactate, using glutamine as a significant source of carbon.

**Figure 4:**
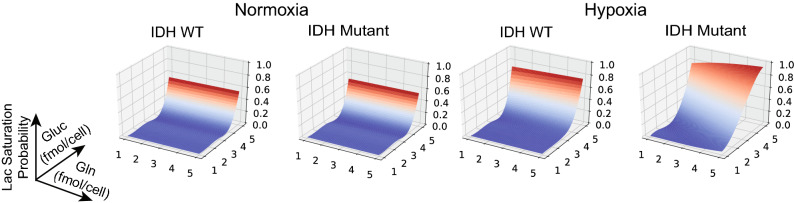
Probability of lactate saturation as a function of initial concentration of nutrients. The PRISM models were instantiated with corresponding phenotype and conditions as in the experimental models. Glucose and glutamine initial concentrations are varied over 1–5 (fmol/cell) and all other species are set to 0. The model checking problem was expressed as computing the probability of reaching a state in which lactate is saturated at a given point in time.

The dependence of lactate on glutamine is better understood by looking at how the probability of lactate saturation—lactate amount reaching the maximum amount set in the model—is affected by the initial concentration of glucose and glutamine. We model check the probability that lactate is saturated and show the results in Fig. 4. The results show that while under normoxia the probability of lactate saturation depends only on the concentration of glucose, under hypoxia and for the $\mathrm {IDH}^\mathrm {mut}$ cell line, the probability also increases with the increase in glutamine concentration.

### Patient-derived phenotypes reveal differences based on the IDH-mutation status

The models constructed with cell line profiling data showed the limitations of the biological models based on overexpression of mutant IDH. We set out to explore whether patient data would reveal a different story for $\mathrm {IDH}^\mathrm {mut}$ metabolic profiles. In particular, we hypothesized that the metabolic profiles of IDH-mutant gliomas differ from the wild-type ones, and that of normal brain.

The lack of direct measures of metabolic activity in patient samples precludes direct modeling of their metabolism. Similarly, proteomic and phospho-proteomic profiling remains sparse, or has limited coverage (for example, TCGA reverse phase protein array profiling was performed on the majority of the samples, but only covered a combined 211 proteins/phsospho-proteins) therefore being of limited utility in our context. However, transcriptomic profiling has been extensively applied in patient samples, and can provide estimates on relative differences in metabolic activity of specific enzymes between phenotypes based on the differences in expression of the genes encoding those enzymes^17^.

Since no absolute rates can be determined based on transcriptomic information, and given that the $\mathrm {IDH}^\mathrm {wt}$ MFA model expressed the classical behavior of the Warburg phenotype, we assumed that this model closely recapitulates the known Warburg phenotype of IDH wild type gliomas. We then estimated the relative differences of this phenotype with the patient $\mathrm {IDH}^\mathrm {mut}$ and normal brain phenotypes based on relative differences in gene expression (mRNA) data from patients. Similar to Khurshed et al.^17^, we used the fold change of expression for genes coding for key metabolic enzymes as a proxy for the relative rates of the corresponding reactions; when multiple genes encoded the same enzyme or isoenzymes, these genes expression’ was aggregated to obtain differences at the enzyme level.

Cohorts from glioma and normal brain samples from TCGA were constructed as follows: 140 $\mathrm {IDH}^\mathrm {wt}$ GBMs formed the classical Warburg cohort, 419 $\mathrm {IDH}^\mathrm {mut}$ LGGs formed the $\mathrm {IDH}^\mathrm {mut}$ phenotype, and 5 normal (whole organ) brain samples formed the normal phenotype. We excluded $\mathrm {IDH}^\mathrm {wt}$ LGGs and $\mathrm {IDH}^\mathrm {mut}$ GBMs since they represent intermediate phenotypes, as can be seen by their prognosis (see Supplemental Figure S2 for the Kaplan–Meier estimates of survival): $\mathrm {IDH}^\mathrm {wt}$ LGGs had intermediate prognosis between GBMs with $\mathrm {IDH}^\mathrm {wt}$  and GBMs with $\mathrm {IDH}^\mathrm {mut}$, and $\mathrm {IDH}^\mathrm {mut}$ GBMs had intermediate prognosis between $\mathrm {IDH}^\mathrm {wt}$ LGGs and $\mathrm {IDH}^\mathrm {mut}$ LGGs^27^.

**Figure 5:**
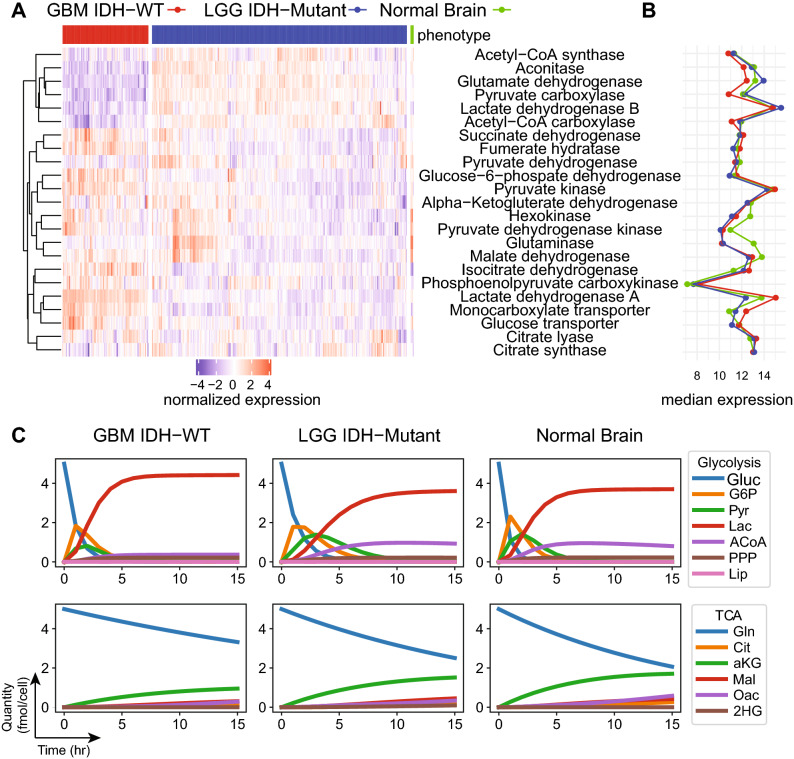
Metabolic phenotypes of patient derived samples. (**A**) Patient-derived mRNA expression data from The Cancer Genome Atlas of genes encoding for TCA and Glycolysis enzymes were processed and aggregated to estimate enzyme levels for three cohorts: Glioblastoma (GBM) samples with IDH-WT phenotype, Lower Grade Glioma (LGG) Samples with IDH-Mutant phenotype, and Normal Brain samples; displayed are across-samples-normalized expression heatmaps. (**B**) Estimated median expression of the enzymes for each cohort. (**C**) Using the experimental model flux rates from Grassian et al. ^14^ as the predicted GBM IDH WT rates, the flux rates for the other two cohorts were estimated using the ratios of the expression between GBM IDH-WT and LGG IDH-Mutant, and between GBM IDH-WT and Normal Brain. The PRISM models were instantiated with corresponding rates. Glucose and glutamine are initiated at maximum quantity 5 (fmol/cell) and all other species at 0.

The differences in expression levels of the key glucose metabolism enzymes and transporters, aggregated at the functional category level, are shown in Table 1 and Fig. 5A, B. (Gene level fold changes and which genes’ expression is aggregated for each enzyme are shown in Supplemental Table S2; the computed flux rates are shown in Supplemental Table S3).

The resulting models were studied as before for behavior over time, as well as how the behavior is affected by initial nutrient concentration (results are summarized in Fig. 5, Supplemental Figures S3, S4). As seen in Fig. 5C, the Warburg phenotypes is considerably more glycolytic than both the normal and $\mathrm {IDH}^\mathrm {mut}$ phenotypes, and has weakened TCA cycle by comparison. The $\mathrm {IDH}^\mathrm {mut}$ phenoytpe is the least efficient among the three phenotypes, both in terms of glucose and glutamine utilization, as well as slower lactate accumulation.

**Table 1:**
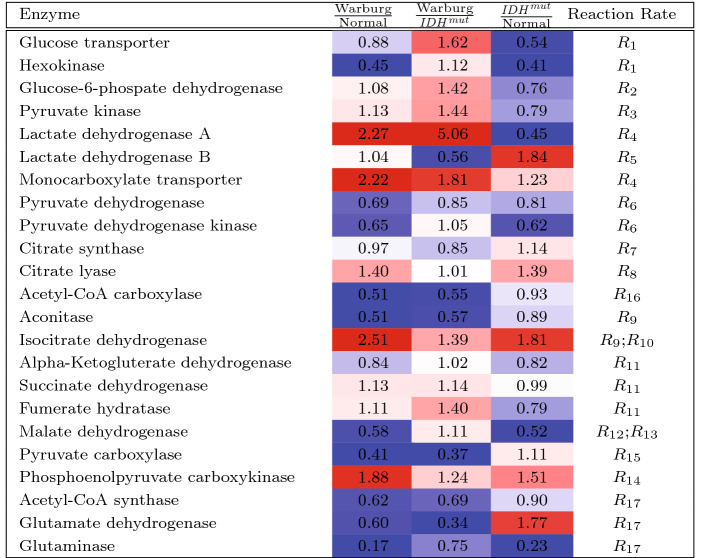
Fold change of metabolic enzymes expression by phenotype

Gene expression data from TCGA cohorts were aggregated at the enzyme/functional category level, and differential expression analysis was performed to assess the relative differences in the patient sample cohorts of GBM IDH wt, LGG IDH mutant, and Normal brain. These fold changes were used as the estimated relative difference in the reaction rates as listed.

## Discussion

Formal computational modeling of metabolic networks can provide insights on the global metabolic state of cancer cells: how they utilize nutrients and to what end. The ability to build models with incomplete information, and the flexibility to vary the initial conditions (nutrient concentration, rates) and add or remove components, makes it possible to perform complex in silico experiments. Crucially, these experiments can offer predictions on how the biological systems might behave when tested or perturbed in analogous ways.

Our models of a metabolic network with components from glucose and glutamine metabolism provided several important insights. First, they showed that biological models in which IDH mutations are induced exogenously do not fully recapitulate the metabolic profile of cells endogenously expressing mutant-IDH. Under normoxia, the MFA models of the isogenic $\mathrm {IDH}^\mathrm {wt}$ and $\mathrm {IDH}^\mathrm {mut}$ cell lines showed a classical Warburg phenotype, with virtually no differences between the two. Under hypoxia, however, the exogenous mutant-IDH had a significant impact on increasing the flux of glutamine-derived metabolites towards lactate production. In addition, our results suggest that profiling of endogenous mutant-IDH cell lines, which are notoriously difficult to grow, is necessary to understand the full impact of IDH mutations on metabolism.

Nevertheless, we showed the utility of integrating MFA data from in vitro experiments with patient-derived mRNA data by predicting the overall metabolic profile of $\mathrm {IDH}^\mathrm {mut}$ gliomas and normal brain based on the relative expression of genes encoding metabolic enzymes. We found that $\mathrm {IDH}^\mathrm {wt}$ glioblastomas are significantly more glycolytic than both normal brain and $\mathrm {IDH}^\mathrm {mut}$ gliomas. This is in line with findings from the literature: Oudard et al.^28^ found that glycolysis in gliomas was elevated to an average of 3-fold compared to normal brain. The accuracy of using mRNA expression as a proxy for protein levels depends on good correlation between mRNA expression and protein levels, which is generally not true. In particular, a model based on mRNA expression data does not account for phosphorylation events, and although differences in the phenotypes could be observed, we expect the differences in enzymatic activities to be even more pronounced because of phosphorylation and posttranslational modifications. Encouragingly, however, a recent study in an ovarian cancer xenograft model found that differentially expressed mRNAs correlate significantly better with their protein product than non-differentially expressed mRNAs^29^.

The normal brain phenotype that we model is based on data obtained at the whole organ level. While at the whole organ level normal brain is glycolytic, a more complex picture has emerged at the cellular level, complicated also from the fact that most experiments at the cellular level are done in vitro on preparations enriched in astrocytes, neurons, or endothelial cells and therefore do not fully recapitulate the in vivo scenario. Neurons rely on oxidative metabolism to meet their high energy needs and produce minimal lactate, whereas astrocytes are highly glycolytic and produce lactate^30^. Lactate and to a lesser degree pyruvate produced by astrocytes are released in the extracellular space to be utilized by the neurons. It has been noted that neurons have the ability to fully oxidize lactate, thereby supporting the role of astrocytes providing lactate as an alternative fuel.

Our models can also help reason about how changing the parameters of the model affects their behavior. An intriguing question is whether formulation of phenotypic shifts (for example reversal of the Warburg phenotype to a more benign non-Warburg phenotype) can be formulated as an optimization problem, and solved to predict which reactions could be targeted to achieve such shifts. Unfortunately, our approach has shed light on the fact that the Warburg phenotype cannot be reversed by simply introducing the IDH1 mutation in the parental glioblastoma phenotype. However, it is possible in this approach to explicitly change the rates of the reactions (simulating knock-down or over-expression experiments) and study the resulting behavior. Affecting the metabolic phenotypes of cancer—driving them towards normal or maladapted phenotypes—has been proposed as a treatment strategy. For example, Poteet et al.^31^ reversed the Warburg effect in glioblastoma through the use of methylane blue in an in vitro glioblastoma model: the reversal was evidenced by an increase in oxygen consumption and reduction of lactate production. The overall effect of reduction in the biosynthesis of intermediates feeding proliferation led to slower proliferation of the glioblastoma cells. Another reason to target the Warburg effect is because this phenotype may be an adaptation to the gradients of hypoxia present in the microenvironment of fast growing tumors, and which may drive reliance of tumor cells on glycolysis for their ATP-production needs^32^.

Other mathematical and computational approaches exist for constructing models of metabolism. Our model checking results are in agreement with the published work by Grassian et al.^14^, who used a deterministic kinetic modeling approach, therefore serving as validation of the model checking approach as a suitable alternative to the kinetic deterministic modeling of metabolism. Computational approaches in addition to model checking include Petri Nets, process calculi, and hybrid approaches^12^. Baldan et al.^33^, for example, review modeling of metabolic pathways using Petri Nets. The Petri Net representation closely resembles the graphical representations of metabolic pathways as networks: tokens flow in a network of connected places according to transition firing rules that, depending on the flavor of the formalism, may be governed by quantitative rules like timed rates, or stochasticity. Petri Nets are amenable to qualitative structural analyses, which in a biological setting can be used, for example, to establish their continuous operation/presence of steady states, or quantitative analyses that are often synonymous with simulation^33^. Reddy et al.^34^, and Hardy and Robillard^35^ modeled glycolysis and pentose phosphate pathways in erythrocytes using several flavors of Petri Nets, and showed (upper) boundaries for certain molecular species, conservation properties, and situations leading to deadlocking of the system. Finally, Fisher and Henzinger^12^ overview the fundamental differences between mathematical and computational approaches and the appeal of choosing one approach over the other given a biological modeling task. They explain that mathematical models are difficult to obtain when relationships between the variables change over time and depend on certain qualitative events (such as a saturation concentration being reached), making the computational approaches effective in such contexts. Moreover, computational approaches allow for specification at different levels of abstraction, and can therefore be useful even when a process that is being described is not understood in detail.

We abstracted over compartment information and therefore did not model the shuttling of the molecules in and out of the cytosol or mitochondria. However, metabolism in cancers is often regulated through regulation of transporters of molecules and their isoforms. For example, mitochondral pyruvate carrier (MPC), which carries pyruvate into mitochondria, is composed mainly of two isoforms: MPC1 and MPC2. Lower levels of MPC1 (whether by deletion of its genomic locus or lower expression) are associated with poorer prognosis in gliomas and stronger Warburg effect^36^. Possible extensions of the model that can be handled within the PRISM framework could include explicit modeling of compartments and molecular transporters. A more complete picture of metabolism will also inevitably require modeling how the metabolic pathways interface and interact with other major signaling pathways.

## Supplementary Information

Supplementary Information.

## Data Availability

Metabolic flux analysis rates data were obtained from the publication of Grassian et al.^14^. Public TCGA expression data were downloaded and processed using R’s TCGAWorkflow^21^. The constructed model has been deposited to the public Biomodels repository under identifier MODEL2207180001 (https://www.ebi.ac.uk/biomodels/MODEL2207180001).
