# Supplementary material for: Probabilistic model checking of cancer metabolism
Source: Sci Rep. 2022 Nov 7;12:18870. doi: 10.1038/s41598-022-21846-5 (PMC9640632; doi:10.1038/s41598-022-21846-5)
Supplement: Supplementary file 1 — Supplementary Information. [file 41598_2022_21846_MOESM1_ESM.pdf]

# Supplemental Information

# Probabilistic Model Checking of Cancer Metabolism

**Meir R. Friedenberg, Adrian Lita, Mark R. Gilbert, Mioara Larion, and Orieta Celiku**

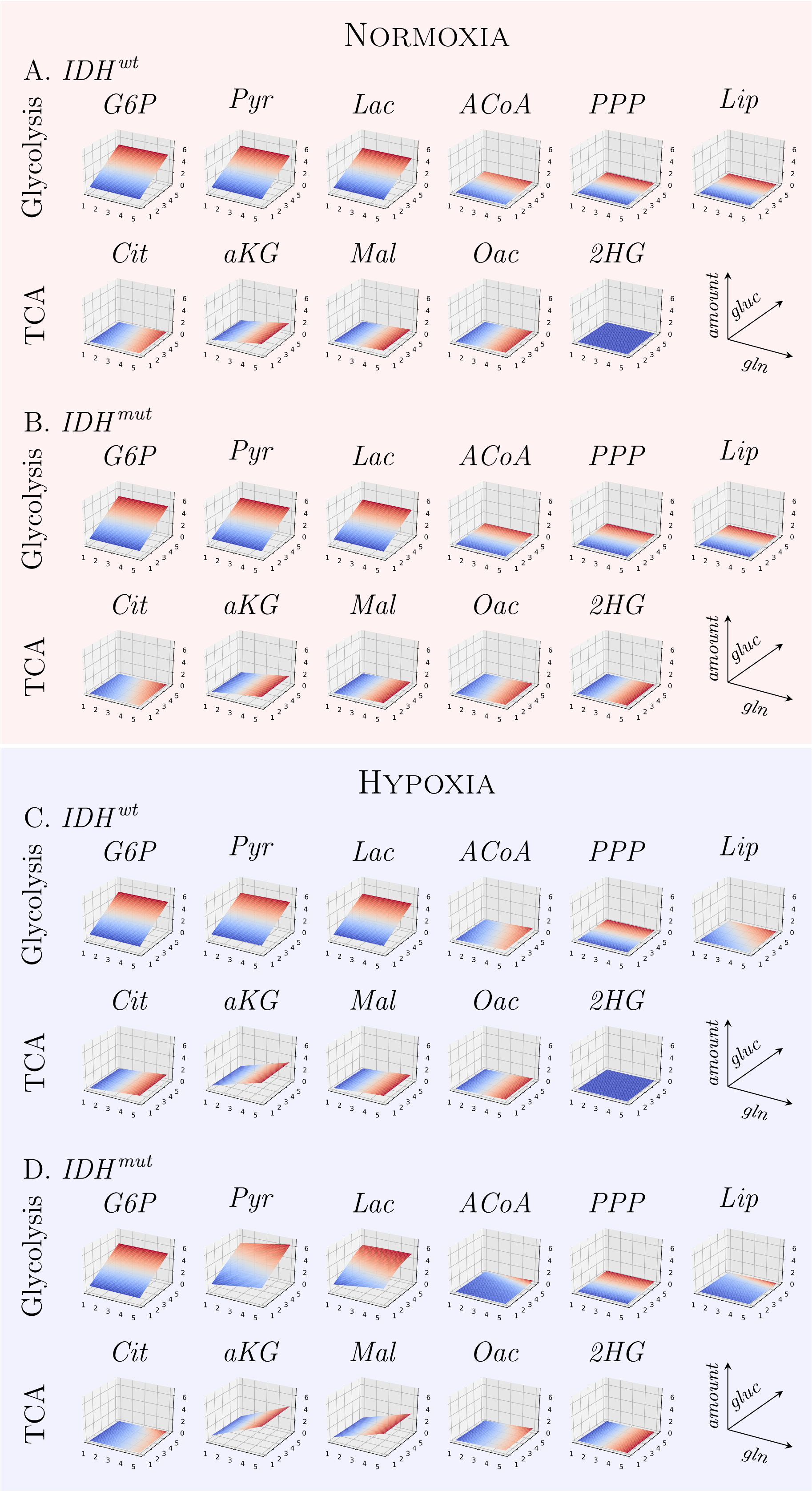


**Figure S1.** Effect of Nutrient Concentration on Cumulative Metabolite Quantities under Normoxia and Hypoxia Conditions. Effect of simultaneously varying initial glucose concentration (from 1 to 5 (fmol/cell)) and initial glutamine (from 1 to 5 (fmol/cell)) on cumulative metabolites produced. The PRISM models were instantiated with corresponding phenotype and condition rates as in the experimental models. The model checking problem was expressed as characterizing the cumulative quantity of molecular species from initialization up to a moment in time.

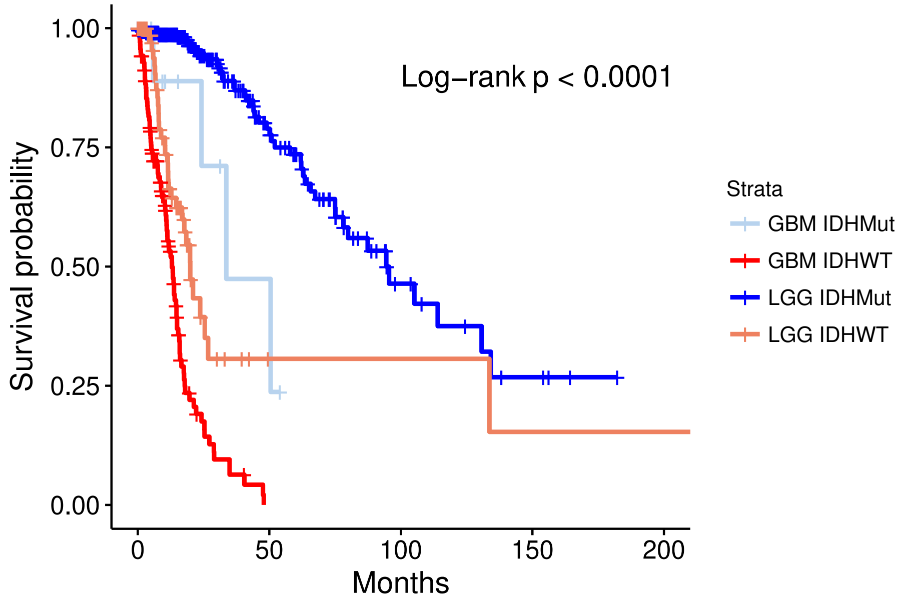


**Figure S2.** Kaplan-Meir Overall Survival of TCGA Glioma Patients. To select the patient samples that are most representative of the glioma IDH wt and IDH mutant phenotypes, we excluded among the TCGA glioblastoma study samples those that had IDH mutant status, and excluded from the lower grade glioma study samples those that had IDH wt status. As can be seen by the survival curves, these samples represent intermediate phenotypes of disease aggressivity, as their overall survival falls between the two selected cohorts, and therefore we hypothesized that their metabolic phenotypes would also fall in between the classical Warburg phenotype of the IDH wt samples, and the classical IDH mutant phenotype.

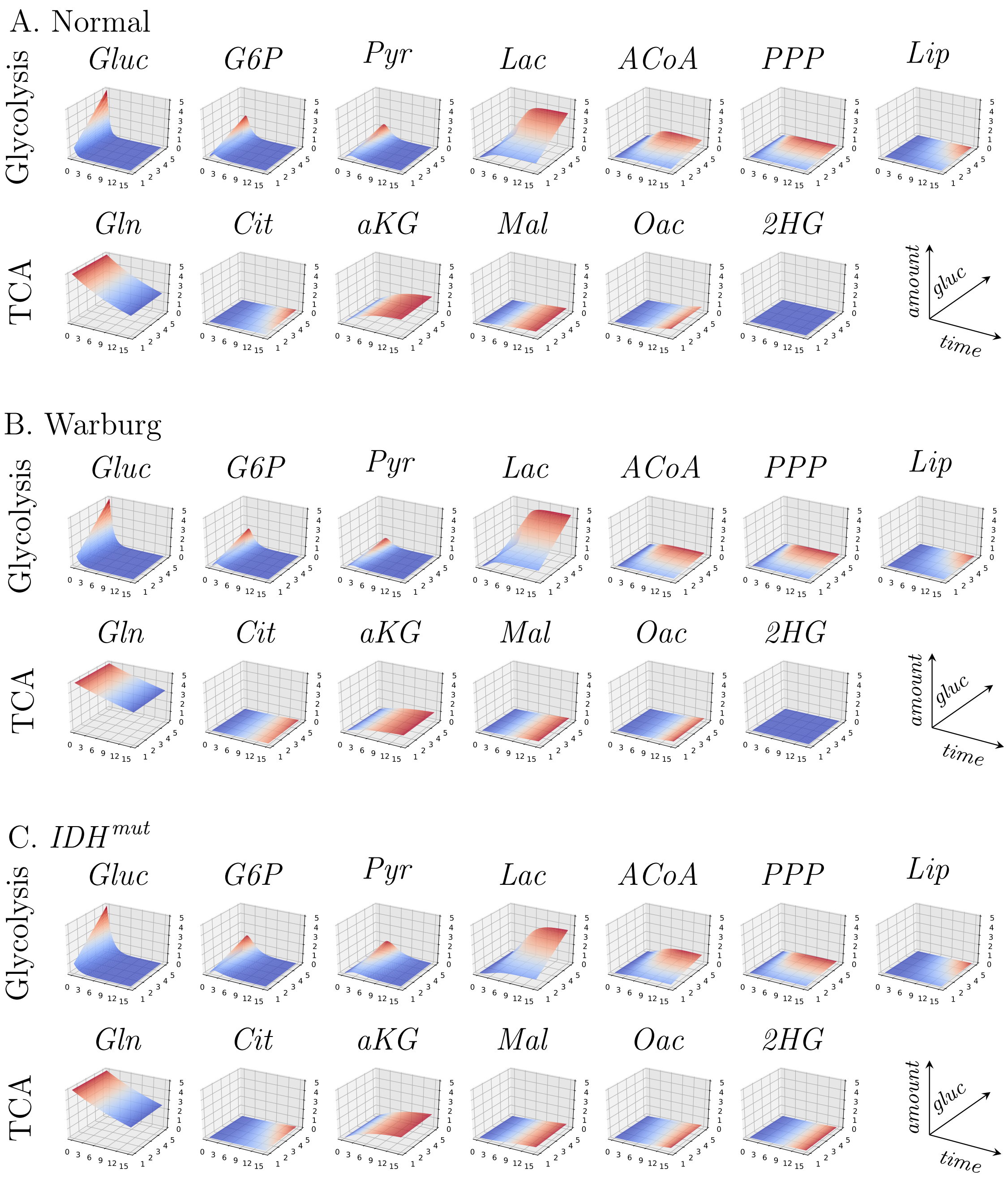


**Figure S3.** Effect of Varying Initial Concentration of Glucose under Normoxia and Hypoxia Conditions. The models derived from the integration of flux analysis data and patient derived relative rates were model checked against the transient quantitites of metabolites produced, to assess predicted effect of varying initial concentration of glucose.

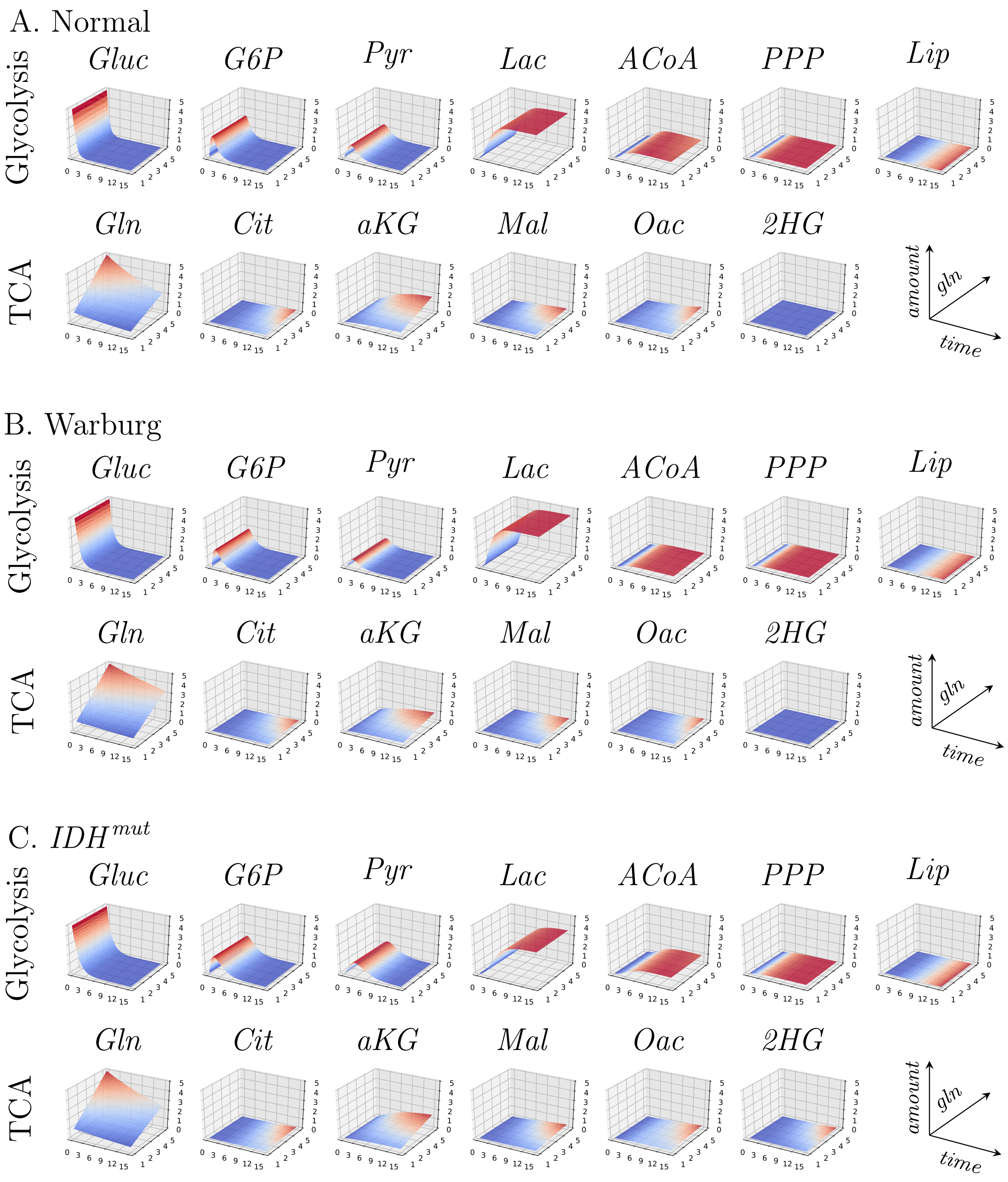


**Figure S4.** Effect of Varying Initial Concentration of Glutamine under Normoxia and Hypoxia Conditions. The models derived from the integration of flux analysis data and patient derived relative rates were model checked against the transient quantitites of metabolites produced, to assess predicted effect of varying initial concentration of glutamine.

| Reaction | Rate | $IDH^{wt}$ Normoxia | $IDH^{mut}$ Normoxia | $IDH^{wt}$ Hypoxia | $IDH^{mut}$ Hypoxia |
|---|---|---|---|---|---|
| Gluc→GCP | $R_1$ | 1.0000 | 1.0073 | 2.4897 | 2.5347 |
| G6P→PPP | $R_2$ | 0.0432 | 0.0539 | 0.1102 | 0.1362 |
| G6P→Pyr | $R_3$ | 0.9570 | 0.9533 | 2.3795 | 2.3984 |
| Pyr→Lac | $R_4$ | 1.6499 | 1.5728 | 4.6856 | 4.6823 |
| Lac→Pyr | $R_5$ | 0.0001 | 0.0001 | 0.0001 | 0.0001 |
| Pyr→ACoA | $R_6$ | 0.1357 | 0.1116 | 0.1092 | 0.0721 |
| ACoA+Oac→Cit | $R_7$ | 0.1357 | 0.1116 | 0.1092 | 0.0721 |
| Cit→ACoA+Oac | $R_8$ | 0.0948 | 0.0721 | 0.1135 | 0.0443 |
| Cit→aKG | $R_9$ | 0.0409 | 0.0395 | 0.0000 | 0.0278 |
| aKG→Cit | $R_{10}$ | 0.0082 | 0.0050 | 0.0252 | 0.0031 |
| aKG→Mal | $R_{11}$ | 0.0681 | 0.0564 | 0.0601 | 0.1597 |
| Mal→Oac | $R_{12}$ | 0.1296 | 0.1052 | 0.0990 | 0.0400 |
| Oac→Mal | $R_{13}$ | 0.0001 | 0.0001 | 0.0001 | 0.0001 |
| Mal→Pyr | $R_{14}$ | 0.0222 | 0.0148 | 0.0612 | 0.1588 |
| Pyr→Oac | $R_{15}$ | 0.0061 | 0.0064 | 0.0102 | 0.0321 |
| ACoA→Lip | $R_{16}$ | 0.0001 | 0.0001 | 0.0001 | 0.0001 |
| Gln→aKG | $R_{17}$ | 0.0273 | 0.0232 | 0.0643 | 0.1334 |
| aKG→2HG | $R_{18}$ | 0.0000 | 0.0063 | 0.0000 | 0.0015 |

**Table S1.** Experimental Flux Rates under Normoxia and Hypoxia Conditions. These are the experimental metabolic flux rates from Grassian et al.[14] which were used to construct the initial PRISM models.

| Enzyme | Symbol | $\frac{\text{Warburg}}{\text{Normal}}$ | $\frac{\text{Warburg}}{IDH^{mut}}$ | $\frac{IDH^{mut}}{\text{Normal}}$ |
|---|---|---|---|---|
| Glucose transporter | SLC2A1 | 1.90 | 1.62 | 1.17 |
| | SLC2A2 | 12.63 | 3.98 | 3.18 |
| | SLC2A3 | 0.45 | 2.51 | 0.18 |
| Hexokinase | HK1 | 0.31 | 0.93 | 0.33 |
| | HK2 | 3.31 | 2.17 | 1.52 |
| | HK3 | 14.42 | 5.25 | 2.75 |
| Glucose-6-phospate dehydrogenase | G6PD | 1.21 | 1.55 | 0.78 |
| Pyruvate kinase | PKLR | 0.13 | 0.31 | 0.42 |
| | PKM2 | 1.22 | 1.56 | 0.78 |
| Lactate dehydrogenase A | LDHA | 2.50 | 5.60 | 0.45 |
| Lactate dehydrogenase B | LDHB | 1.11 | 0.59 | 1.88 |
| Monocarboxylate transporter | SLC16A1 | 2.20 | 1.61 | 1.36 |
| | SLC16A3 | 5.11 | 4.48 | 1.14 |
| Pyruvate dehydrogenase | PDHA1 | 0.67 | 0.85 | 0.80 |
| | PDHB | 0.92 | 1.03 | 0.89 |
| | PDHX | 0.61 | 0.77 | 0.79 |
| Pyruvate dehydrogenase kinase | PDK1 | 2.03 | 3.33 | 0.61 |
| | PDK2 | 0.47 | 0.74 | 0.63 |
| | PDK3 | 1.13 | 2.26 | 0.50 |
| Citrate synthase | CS | 1.04 | 0.90 | 1.15 |
| Citrate lyase | ACLY | 1.55 | 1.10 | 1.41 |
| Acetyl-CoA carboxylase | ACACA | 0.58 | 0.55 | 1.07 |
| | ACACB | 0.44 | 1.02 | 0.43 |
| Aconitase | ACO1 | 1.47 | 0.89 | 1.65 |
| | ACO2 | 0.46 | 0.55 | 0.83 |
| Isocitrate dehydrogenase | IDH1 | 6.56 | 2.01 | 3.27 |
| | IDH2 | 1.44 | 1.05 | 1.38 |
| Alpha-Ketogluterate dehydrogenase | OGDH | 0.91 | 1.09 | 0.83 |
| Succinate dehydrogenase | SDHA | 0.69 | 0.77 | 0.89 |
| | SDHB | 1.22 | 1.44 | 0.85 |
| | SDHC | 1.88 | 1.59 | 1.18 |
| | SDHD | 1.95 | 1.35 | 1.45 |
| Fumerate hydratase | FH | 1.20 | 1.49 | 0.81 |
| Malate dehydrogenase | MDH1 | 0.36 | 0.95 | 0.38 |
| | MDH2 | 1.38 | 1.43 | 0.97 |
| Pyruvate carboxylase | PC | 0.45 | 0.39 | 1.15 |
| Phosphoenolpyruvate carboxykinase | PCK1 | 1.34 | 0.76 | 1.76 |
| | PCK2 | 2.05 | 1.36 | 1.51 |
| Acetyl-CoA synthase | ACSS1 | 0.63 | 0.59 | 1.07 |
| | ACSS2 | 0.80 | 1.09 | 0.74 |
| Glutamate dehydrogenase | GLUD1 | 0.63 | 0.34 | 1.83 |
| | GLUD2 | 0.70 | 0.44 | 1.60 |
| Glutaminase | GLS | 0.19 | 0.81 | 0.23 |
| | GLS2 | 0.05 | 0.31 | 0.16 |

**Table S2.** Fold Change of Metabolic Enzyme Gene Expression by Phenotype. Detailed listing of which genes's expression was used to obtain estimates of the expression of the listed enzymes, and the resulting fold changes, as expression of the relative differences in metabolic activities.

| Reaction | Rate | Normal | Warburg | $IDH^{mut}$ |
|---|---|---|---|---|
| Gluc→GCP | $R_1$ | 1.5038 | 1.0000 | 0.7299 |
| G6P→PPP | $R_2$ | 0.0400 | 0.0432 | 0.0304 |
| G6P→Pyr | $R_3$ | 0.8469 | 0.9570 | 0.6646 |
| Pyr→Lac | $R_4$ | 0.7349 | 1.6499 | 0.4803 |
| Lac→Pyr | $R_5$ | 0.0001 | 0.0001 | 0.0002 |
| Pyr→ACoA | $R_6$ | 0.2025 | 0.1357 | 0.1428 |
| ACoA+Oac→Cit | $R_7$ | 0.1399 | 0.1357 | 0.1596 |
| Cit→ACoA+Oac | $R_8$ | 0.0677 | 0.0948 | 0.0939 |
| Cit→aKG | $R_9$ | 0.0271 | 0.0409 | 0.0417 |
| aKG→Cit | $R_{10}$ | 0.0033 | 0.0082 | 0.0059 |
| aKG→Mal | $R_{11}$ | 0.0663 | 0.0681 | 0.0574 |
| Mal→Oac | $R_{12}$ | 0.2234 | 0.1296 | 0.1168 |
| Oac→Mal | $R_{13}$ | 0.0002 | 0.0001 | 0.0001 |
| Mal→Pyr | $R_{14}$ | 0.0118 | 0.0222 | 0.0179 |
| Pyr→Oac | $R_{15}$ | 0.0149 | 0.0061 | 0.0165 |
| ACoA→Lip | $R_{16}$ | 0.0002 | 0.0001 | 0.0002 |
| Gln→aKG | $R_{17}$ | 0.0589 | 0.0273 | 0.0460 |
| aKG→2HG | $R_{18}$ | 0.0000 | 0.0000 | 0.0062 |

**Table S3.** Computed Rates by Phenotype. The rates of the IDH wt cell line under normoxia from Grassian et al.[14] were assumed to be representative of the classical Warburg phenotype, which in turn is the known phenotype of IDH wild type glioblastomas. These rates were multiplied with the fold changes of the enzyme expressions to derive estimates of the rates for Normal brain, and the IDH mutant gliomas, and were used to instantiate the integrated patient derived models.
